# Limits to Hole Mobility and Doping in Copper Iodide

**DOI:** 10.1021/acs.chemmater.3c01628

**Published:** 2023-10-25

**Authors:** Joe Willis, Romain Claes, Qi Zhou, Matteo Giantomassi, Gian-Marco Rignanese, Geoffroy Hautier, David O. Scanlon

**Affiliations:** † Department of Chemistry, University College London, 20 Gordon Street, London WC1H 0AJ, U.K.; ‡ Thomas Young Centre, University College London, Gower Street, London WC1E 6BT, U.K.; § UCLouvain, Institute of Condensed Matter and Nanosciences (IMCN), Chemin des Étoiles 8, Louvain-la-Neuve B-1348, Belgium; ∥ Thayer School of Engineering, Dartmouth College, Hanover, New Hampshire 03755, United States; ⊥ School of Chemistry, University of Birmingham, Edgbaston, Birmingham B15 2TT, U.K.

**Keywords:** copper, charge transport, holes, mobility, scattering, CuI, defects, density functional theory/DFT

## Abstract

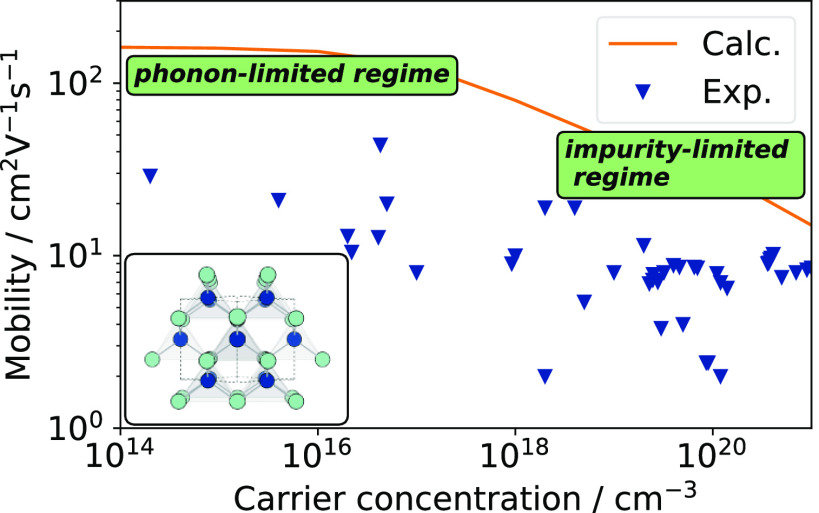

Over one hundred years have passed since the discovery of the p-type transparent conducting material copper iodide, predating the concept of the “electron–hole” itself. Supercentenarian status notwithstanding, little is understood about the charge transport mechanisms in CuI. Herein, a variety of modeling techniques are used to investigate the charge transport properties of CuI, and limitations to the hole mobility over experimentally achievable carrier concentrations are discussed. Poor dielectric response is responsible for extensive scattering from ionized impurities at degenerately doped carrier concentrations, while phonon scattering is found to dominate at lower carrier concentrations. A phonon-limited hole mobility of 162 cm^2^ V^–1^ s^–1^ is predicted at room temperature. The simulated charge transport properties for CuI are compared to existing experimental data, and the implications for future device performance are discussed. In addition to charge transport calculations, the defect chemistry of CuI is investigated with hybrid functionals, revealing that reasonably localized holes from the copper vacancy are the predominant source of charge carriers. The chalcogens S and Se are investigated as extrinsic dopants, where it is found that despite relatively low defect formation energies, they are unlikely to act as efficient electron acceptors due to the strong localization of holes and subsequent deep transition levels.

## Introduction

High mobility p-type transparent conducting materials (TCMs) have eluded researchers for decades. The unlikely bedfellows of good optical transparency (a wide band gap >3 eV and above 90% visible transmission), high valence band dispersion (typically requiring carrier effective mass < 0.5*m*_e_), and correct point defect chemistry (facile and controllable generation of electron–holes up to 10^21^ cm^–3^, depending on applications) prove difficult to unite. In fact, these strict requirements preclude the vast majority of materials from ever displaying transparent p-type conducting properties. Early efforts focused on the development of p-type oxides, such as CuAlO_2_,^1^ attempting to mimic the wide optical band gap of the n-type transparent conducting oxides. However, CuAlO_2_ and other delafossite materials are plagued by low mobility and conductivity due to the polaronic nature of the holes generated in these systems, which are bound to Cu states at the valence band maximum.^2−13^

Several other oxides have been considered as p-type transparent conductors, with varying degrees of success: Li-doped NiO can reach hole concentrations on the order of 1 × 10^21^ cm^–3^ and conductivity up to 11 S cm^–1^, but mobility is less than 0.05 cm^2^ V^–1^ s^–1^ and transmission drops to 50% upon doping;^14^ SnO is a reasonably good p-type TCM, with mobility around 7 cm^2^ V^–1^ s^–1^ at 1 × 10^17^ cm^–3^ carriers, and can also be doped n-type, but incurs stability issues due to the presence of Sn(II) [a problem which pervades ternary Sn(II) materials also];^15,16^ quasi-closed shell d^3^ and d^6^ materials such as Sr-doped LaCrO_3_ and the ZnM_2_O_4_ spinels (M = Co, Rh, Ir) have been investigated as TCMs, exploiting the large crystal field splitting between e_g_ and t_2g_ states to engineer transparency, but their mobilities and conductivities routinely fall short of requirements;^17,18^ and Ba_2_BiTaO_6_, which shows excellent mobility (up to 30 cm^2^ V^–1^ s^–1^) due to strong Bi 6s^2^–O 2p interaction at the valence band, but the carrier density is limited to 1 × 10^14^ cm^–3^.^19,20^ Therefore, research into nonoxides has taken center stage in recent years^21−26^ in the hope that greater valence band delocalization and bond covalency can improve hole mobility while simultaneously generating enough carriers to enable degenerate conductivity.

**Figure 1:**
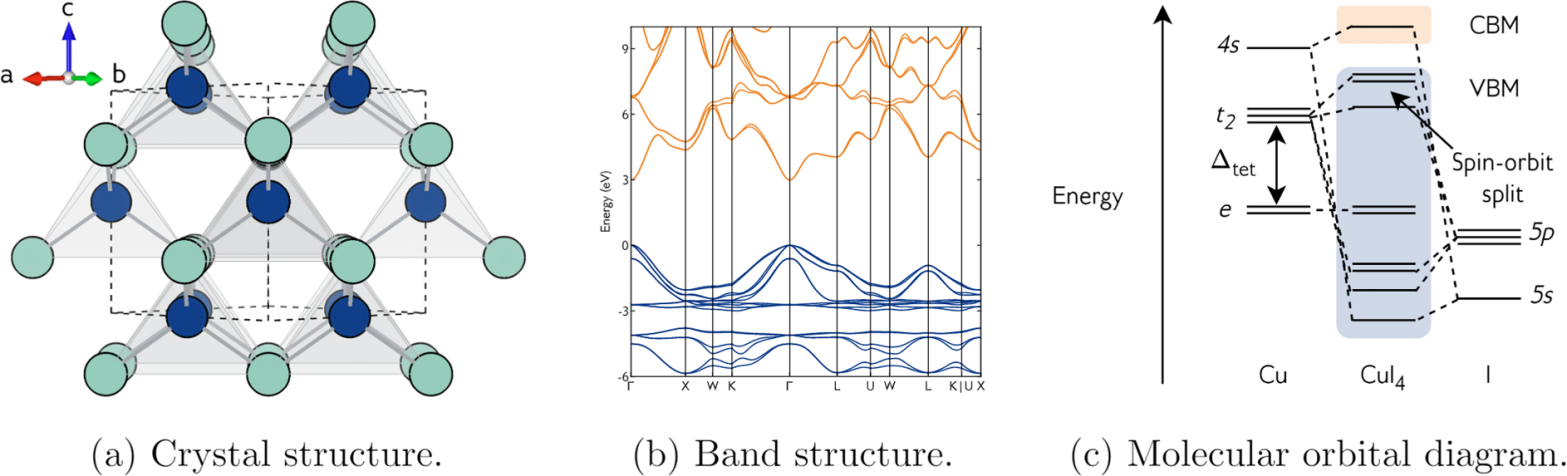
Crystal and electronic structures of CuI. (a) Zincblende crystal structure of CuI, viewed along the face diagonal. Cu and I atoms in blue and green, respectively. Cu atoms have tetrahedral coordination, shown in gray. (b) Electronic band structure of CuI. Calculated using the PBE0 hybrid functional with the inclusion of spin–orbit coupling (SOC). (c) Schematic molecular orbital (MO) diagram of CuI. Upon the inclusion of SOC, further splitting occurs on the t_2_—5p MOs into light and heavy hole channels and a spin–orbit split-off band, as seen in (b)

One such material is copper iodide, CuI, first discovered in 1907 by the “father of transparent conductors” Bädeker,^27^ and now enjoying somewhat of a renaissance. It crystallizes in the cubic zincblende structure below 643 K (Figure 1a), possesses an optical band gap of around 3 eV, displays native p-type conductivity, and consistently shows one of the highest figures of merit, Φ,^a^ for any p-type transparent conductor (over 60,000 MΩ^–1^ for S-doped CuI).^28−30^ It has a disperse, isotropic valence band maximum (Figure 1b), with an average light hole parabolic effective mass of around 0.21*m*_e_, indicative of a reasonably high hole mobility. The valence band maximum is formed by the interaction of Cu 3d t_2_ and I 5p orbitals, while the conduction band minimum is formed by the interaction of Cu 4s with I 5s orbitals,^31,32^ as shown schematically in the molecular orbital (MO) diagram in Figure 1c.
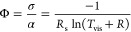
1where *T*_vis_ is transmission and *R*_s_ is the sheet resistance, related to conductivity and thickness *d* by
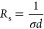
2

In addition to its rather attractive electronic structure, the simplicity of CuI is another strong factor in its revival in popularity. Good quality thin films can be deposited via relatively straightforward techniques such as vaporizing iodine onto thin films of copper (à la Bädeker),^33^ solid- and solution-based iodization reactions,^34−36^ and inkjet printing,^37^ while more advanced techniques such as pulsed laser deposition (PLD),^38^ molecular beam epitaxy (MBE),^39^ and magnetron sputtering are beginning to gain traction.^29^ Stability of films in air and to thermal cycling can be improved by encapsulation with amorphous Al_2_O_3_,^38,40^ to prevent the oxidation of Cu(I) to Cu(II). Single crystal growth of CuI is reported more sporadically and over a much narrower range of charge carrier concentrations.^41,42^ In terms of devices and applications, CuI has been successfully used in thin film transistors (TFTs) with high operational stability and efficiency,^43^ while CuI nanoparticle inks have contributed to improvements in high resolution X-ray imaging technology.^44^ This manufacturing simplicity is further enhanced by its strong compatibility with various n-type oxides (such as ZnO, AgI, BaSnO_3_, and NiI_2_), positioning CuI as an optimal contender for combined n- and p-type applications.^45−51^

Despite this fashionable return to the forefront of p-type TCM research, the charge transport behavior of CuI is poorly understood. Experimental reports show no clear trend between hole mobility and carrier concentration, while little computational work has been undertaken on this subject. In this work, the intrinsic limits of CuI as a high-mobility transparent conductor are investigated from first-principles calculations and state-of-the-art charge carrier transport modeling. The intrinsic defect chemistry and the effects of chalcogen doping are examined to estimate the window of achievable charge carrier concentrations in CuI. The different contributions to the overall scattering rate are analyzed using two separate approaches, where it is found that scattering from phonons and ionized impurities dominate at either end of the charge carrier concentration range, respectively.

## Computational Methodology

Electronic band structure, AMSET inputs, and defect calculations were performed within VASP, a periodic plane-wave code that uses the projector-augmented wave (PAW) method for describing the interaction between core and valence electrons.^52−58^ The explicit electron configurations of the pseudopotentials used were as follows: Cu 3d^10^4s^1^; I 5s^2^5p^5^; S 3s^2^3p^4^; and Se 4s^2^4p^4^. A plane-wave cutoff of 500 eV and a Γ-centered **k**-point mesh of 7 × 7 × 7 were found to converge the total energy to within 1 meV atom^–1^. Structural relaxations were carried out with a plane-wave cutoff of 650 eV to avoid Pulay stress and with a convergence criteria of 0.1 meV atom^–1^. The GGA PBEsol functional was used for all convergence testing and density functional perturbation theory (DFPT)-related inputs,^59^ while the hybrid PBE0 functional was used for electronic structure, defect, and finite differences (FD) calculations.^60^ For all electronic structure calculations, spin–orbit coupling (SOC) effects were explicitly considered due to the presence of heavy I atoms. Considering the size of the spin–orbit split-off in the electronic band structure, the addition of SOC effects via a single-shot electronic structure calculation after structural optimization is crucial to get the correct value of the band gap and relative band edge positions during defect calculations. SOC effects are negligible when computing structural properties, so they were not included during relaxations.

Charge transport properties were calculated by using both ABINIT and the AMSET package. ABINIT uses a fully first-principles approach to calculate phonon-limited mobilities based on an iterative solver to the Boltzmann transport equation (IBTE).^61−64^ The approach used in ABINIT, which is detailed in refs (64) and (65), allow us to achieve a level of performance that is competitive with Wannier-based packages while bypassing the need for Wannier functions altogether. First, our approach takes advantage of the tetrahedron integration scheme to significantly reduce the number of e–ph matrix elements that need to be computed. In addition, we employ a Fourier interpolation of the scattering potentials in **q** space, which includes the accurate treatment of dipole and quadrupole contributions and the exact Kohn–Sham (KS) wave functions are computed only for the **k**-points lying inside a small energy window around the band edges. Here, a convergence is assumed to be reached when three consecutive grids lead to mobilities a maximum 5% away from each other. In CuI, the converged IBTE mobilities were obtained with **k**-meshes of 162 × 162 × 162 and interpolated DFPT scattering potentials and interatomic force constants obtained on a 9 × 9 × 9 coarse **q**-mesh. All the calculations needed to obtain the mobility within ABINIT were done with the GGA PBEsol functional including SOC. The dynamical quadrupoles (Q*) were also included for the scattering potentials and mobilities. However, as the DFPT computation of Q* is still limited to norm-conserving pseudopotentials without nonlinear core corrections (NLCC) and without SOC, a slight deviation of the mobility can be expected but by several orders of magnitude less than doing the calculations without taking the Q* into account.

On the other hand, AMSET solves the linearized Boltzmann transport equation using the relaxation time approximation (RTA). Individual scattering rates were explicitly calculated using material properties, going beyond the constant relaxation time approximation to give a more accurate description of carrier lifetimes. In this study, scattering from polar optical phonons (POP), acoustic deformation potentials (ADP), ionized impurities (IMP), and piezoelectric effects (PIE) were considered, which require deformation potentials, the elastic constant, low- and high-frequency dielectric constants, the polar optical phonon frequency, and the piezoelectric constant. Explicit values (and convergence of these parameters) can be found in the Supporting Information. A dense uniform Γ-centered **k**-point mesh of 14 × 14 × 14 was used to obtain the wave function overlaps for determining scattering rates, and the corresponding band structure was plotted using the SUMO package.^66^ The deformation potentials were calculated from density of states calculations using the same convergence parameters as bulk calculations. The low-frequency dielectric constant, piezoelectric constant, and polar optical phonon frequency were calculated using both DFPT and FD implementations in VASP. The high-frequency dielectric constant was calculated with the PBE0 functional using the independent particle random phase approximation (IP-RPA) optics routine in VASP and converged against the number of empty bands and **k**-point density.

Phonon calculations on CuI were performed with DFPT and ABINIT using PBEsol + SOC and a 9 × 9 × 9 **q**-mesh following the methodology used by Petretto et al.^67^ The structures were relaxed until all the forces on the atoms and the stresses were below 10^–6^ Ha/bohr and 10^–4^ Ha/bohr^3^, respectively, using a plane-wave cutoff of 46 Ha.

Defect calculations were performed with VASP on a 64 atom supercell and with a Γ-centered 2 × 2 × 2 **k**-point mesh. Each defect was fully relaxed using the PBE0 functional, followed by a spin–orbit coupling single-shot calculation to obtain accurate energies. This goes beyond the GGA relaxation plus hybrid single-shot calculations of Graužinytė et al., fully capturing structural distortions caused by charge localization that would otherwise be missed with a semilocal exchange–correlation functional.^68^ The potential energy surface was explored using the ShakeNBreak method.^69,70^ Defect formation energies were obtained in the usual fashion, within the Lany–Zunger formalism^71−74^
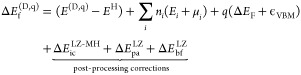
3

The transition level diagrams and self-consistent Fermi level were calculated using the PY-SC-FERMI package.^75,76^

The EFFMASS package was used to analyze the transport effective mass.^77,78^

## Results

### Crystal and Electronic Structure

The structural properties (lattice parameter, lattice angle, and Cu–I bond length) of CuI are summarized in Table 1, where good agreement is found between the calculated values and both the experimental and computational literature. The PBEsol lattice parameters are slightly underestimated compared to room temperature neutron powder diffraction measurements by Keen and Hull,^79^ while the PBE0 results show closer agreement. Low-temperature diffraction data are scarcely reported but are expected to yield lattice parameters closer to the PBEsol results. Thin film CuI often displays slightly larger lattice parameters (6.06 Å from Moditswe et al.)^80^ owing to tensile strain between the film and substrate.

**Table 1:** Structural and Band Structure Properties of CuI

parameter	Exp^79^	PBE0	PBE0 lit^32^	PBEsol
*a*/Å	6.05	6.08	6.07	5.95
α/°	90.0	90.0	90.0	90.0
Cu–I/Å	2.57	2.63	2.62	2.58
band gap/eV	2.95^81^	2.99	2.97	0.95
SOC splitting energy/eV	0.64^82^	0.63		0.47
hole conductivity effective mass/*m*_0_		0.61		0.72

As shown in Table 1, a direct band gap of 2.99 eV at Γ is calculated with the PBE0 functional including spin–orbit coupling effects (PBE0 + SOC, Figure 1b), which is in good agreement with experimental values obtained from a variety of measurements: pressure-dependent optical absorption from Ves et al., 2.95 eV;^81^ transmission spectra from Storm et al., 3.11 eV.^38^ This also matches well previous hybrid DFT calculations, with Yu et al. most recently reporting a value of 2.97 eV.^32^ On the other hand, the direct band gap computed with PBEsol is severely underestimated, as is often the case in semilocal DFT. The hole conductivity effective mass (or the transport mass) determined from PBE0 calculations is slightly lower than that from PBEsol. Semilocal DFT functionals often struggle to accurately describe localized d states (regardless of electronic occupation) without the inclusion of the Hubbard *U* parameter, so a mismatch in the transport effective mass is not unexpected.

**Figure 2:**
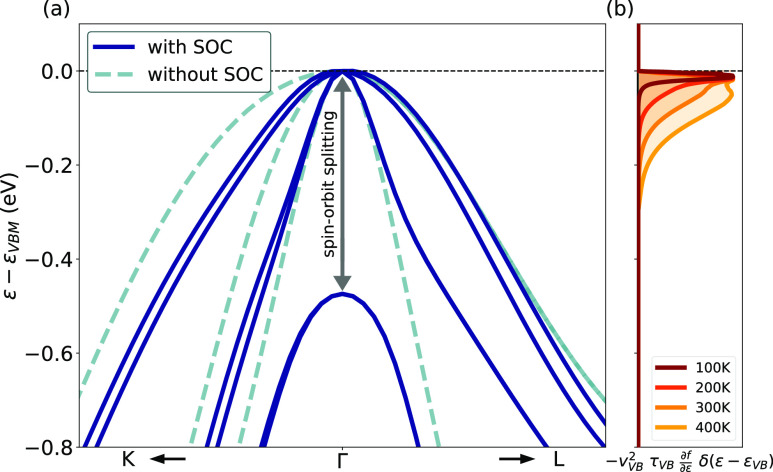
(a) Top of the valence bands of CuI around Γ with and without SOC as computed with the PBEsol functional. (b) The normalized function , convoluted with δ(ε – ε_VB_) in the valence band at different *T*. In this expression, *v*_VB_ and τ_VB_ stand for the carrier velocity and lifetime in the valence band (VB),  is the derivative of the Fermi–Dirac distribution function with respect to the energy and δ the Dirac delta function. By integration of this function, relaxation time approximation (RTA) hole mobility is obtained.

In CuI, the inclusion of SOC has a direct effect on the band edges. Figure 2a illustrates that without SOC, the valence band maximum (VBM) of CuI has three degenerate bands. The addition of SOC results in the lifting of the degeneracy, with one of the bands (the split-off band) dropping in energy, a common feature of zincblende semiconductors. As for the band gap, PBEsol tends to undervalue the spin–orbit splitting at the Γ-point of the VBM which is around 470 meV, whereas the value computed with PBE0 is around 630 meV, in excellent agreement with the value of 640 meV determined experimentally by Blacha et al. using hydrostatic pressure-dependent thin-film absorption.^82^

### Charge Transport

Two methods are used to simulate charge transport in CuI: an iterative Boltzmann transport equation (IBTE) solution to calculate phonon-limited mobilities using the ABINIT code;^61−64^ and a phenomenological model (AMSET) that calculates scattering rates for ADP, PIE, IMP, and POP scattering, utilizing low-cost inputs from first-principles calculations using the VASP code.^52,53,55−58,83^ While IBTE provides a high-quality representation of transport properties, AMSET complements the analysis by evaluating the effects of impurities and also by providing an additional analysis of the phonon scattering mechanisms by decoupling the different contributions. AMSET also offers the advantage of using hybrid functionals, which are difficult to employ in fully first-principles IBTE calculations due to the computational cost and absence of implementations able to compute electron–phonon (e–ph) quantities within DFPT. The methodology for each approach is outlined in the Supporting Information.

#### Iterative Boltzmann Transport Equation

The lifting of degeneracy resulting from the addition of SOC has a clear impact on the hole transport. Indeed, for transport in semiconductors, only energies near the top of the VBM are relevant.^78^ As depicted in Figure 2b, the function , which once integrated provides direct access to the RTA hole mobility, quickly approaches zero for energies further from the Fermi level. This means that only the electronic states covered by this function participate in hole transport. As a result, the inclusion of SOC in CuI leads to the complete removal of a scattering channel as the split-off band is no longer included in the energy windows responsible for transport, even at higher temperatures. Using ABINIT, we obtain a converged IBTE mobility of 162 cm^2^ V^–1^ s^–1^ at 300 K with SOC and dynamical quadrupoles (Q*) using 162 × 162 × 162 **k**- and **q**-point grids (refer to Figure S2). The inclusion of the SOC leads to an enhancement in mobility by removing a scattering channel. Note that, despite the increase in mobility due to the effect of SOC on the electronic bands, its effect on phonons remains negligible on both phonon band structure and mobility, as shown in Figures S1 and S2. The integration of Q*, the next order of correction to dynamical dipoles, in the computation is also necessary to obtain accurate results,^63,64^ preventing an error of about 15% in the case of CuI.

**Figure 3:**
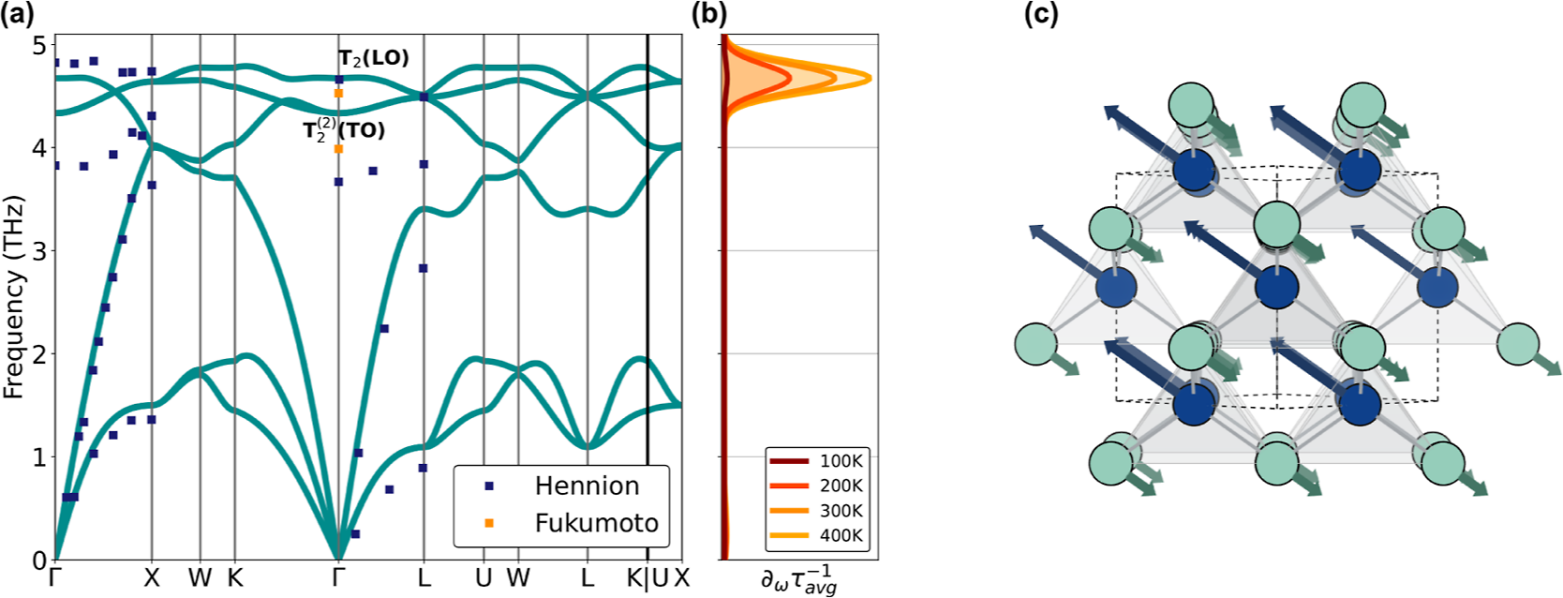
(a) CuI phonon dispersion computed with SOC and the PBEsol functional with experimentally reported frequencies overlaid.^84,85^ (b) Spectral decomposition of the hole scattering rates as a function of frequency at different temperatures. (c) Structure of CuI showing the atomic displacements corresponding to the longitudinal optical phonon mode at the Γ point (toward the *X* direction), viewed along the face diagonal; Cu and I atoms in blue and green, respectively.

The DFPT phonon dispersion of CuI is shown in Figure 3a and shows good agreement with the experimental frequencies reported in the literature.^84,85^ The minor disparity, such as for the LO-TO splitting, may be attributed to the challenge of precisely capturing the dielectric constants in CuI, as discussed in the next section. This plot is accompanied by the corresponding spectral decomposition of the hole scattering rates at different T (Figure 3b), and shows that the high-frequency longitudinal optical mode T_2_ (LO) is the main contributor to the e–ph scattering in CuI. Although the spectral decomposition encompasses both short- and long-range phonons, transport in CuI is mainly influenced by the latter ones (near Γ). This is due to the unique and curvy pocket present at the VBM of the electronic band structure of the material, allowing only transitions with a small momentum transfer **q**. Figure 3c represents the T_2_ (LO) phonon mode of CuI at Γ (toward the *X* direction) with the Cu and I atoms moving in phase opposition with twice the displacement amplitude for Cu. The relative contribution to scattering increases with *T* due to the growing number of phonons that are thermally excited, as evidenced by the area under the curve of Figure 3b expanding from 100 to 400 K. This results in a decrease of the e–ph IBTE mobility with temperature, as shown in Figure S3.

#### AMSET

**Figure 4:**
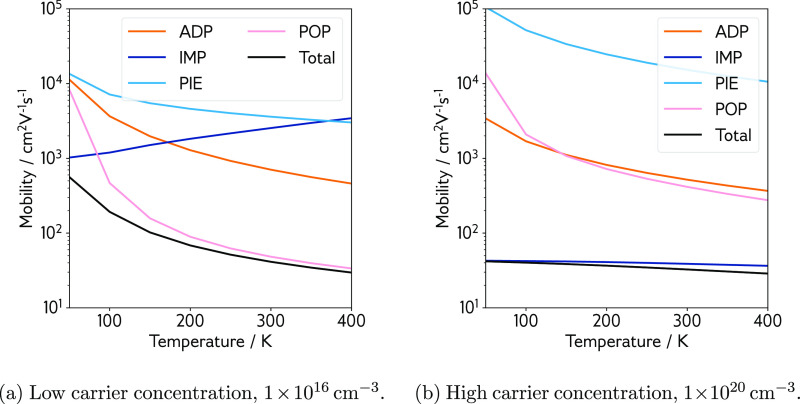
CuI hole mobility as a function of the temperature at two carrier concentrations. Colored lines represent mobility contributions from each type of scattering: ADP is acoustic deformation potential scattering (orange); IMP is ionized impurity scattering (dark blue); PIE is piezoelectric scattering (light blue); POP is polar optical phonon scattering (pink); and total is the reciprocal sum of these contributions (black). (a) Low carrier concentration, 1 × 10^16^ cm^–3^. (b) High carrier concentration, 1 × 10^20^ cm^–3^.

Figure 4 shows the hole mobility calculated with AMSET at low and high carrier concentrations. The mobility is split by scattering mechanism, and the total mobility is plotted as the reciprocal sum of each component (via Matthiessen’s rule). At a carrier concentration of 1 × 10^16^ cm^–3^ (Figure 4a), scattering from POP is predicted to dominate the hole mobility, yielding a total room temperature hole mobility of 41.3 cm^2^ V^–1^ s^–1^. Scattering from ADP, PIE, and IMP is essentially negligible at low concentrations compared to POP scattering. The piezoelectric constant calculated from DFPT is in excellent agreement with the experiment (0.10 C m^–2^ against 0.13 C m^–2^)^86^ and confirms that PIE scattering does not compromise the mobility despite the noncentrosymmetric inversion native to the zincblende crystal structure.

**Figure 5:**
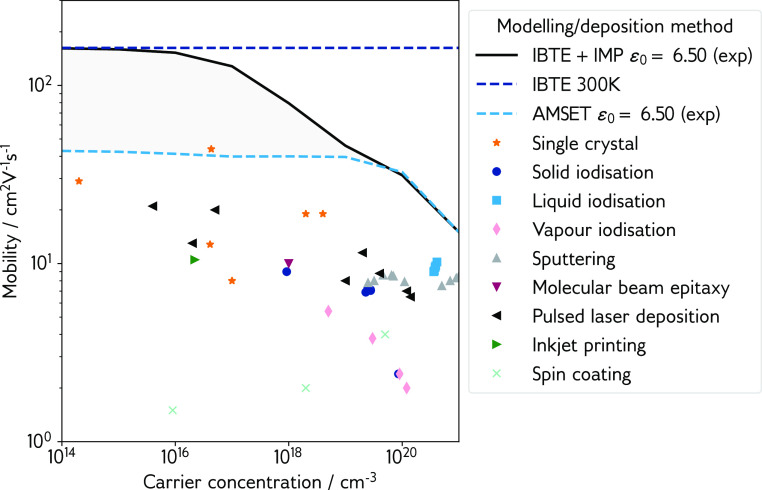
Experimental hole mobility as a function of carrier concentration, broken down by the deposition method. Experimental data are reported in refs (29), (30), (33)–^35^, (37)–^42^, (95)–^96^^97^^98^. IBTE and AMSET drift mobilities overlaid as dashed lines; IBTE + IMP mobility  overlaid as a filled black line; gray shaded region denotes space between AMSET-estimated upper limit and IBTE + IMP-estimated upper limit.

Moving to a higher carrier concentration of 1 × 10^20^ cm^–3^ (Figure 4b), the effects of both POP and PIE scattering are diminished further, while ionized impurities begin to control the charge transport behavior in CuI—a room-temperature hole mobility of 32.6 cm^2^ V^–1^ s^–1^ is predicted. IMP (and indeed POP scattering in the AMSET implementation) scattering is largely determined by the value of the static dielectric response of a material, i.e., its ability to screen electric charge. For CuI, calculated dielectric constants range from 5.27 to 8.85, dependent on the flavor of DFT functional and the method used to calculate the high- and low-frequency responses, and are shown in Table 2. Meanwhile, an experimental dielectric response of 6.5 is reported by Hanson et al.^86^ Accurate calculation of the dielectric response in tetrahedral semiconductors is benchmarked by Skelton et al.,^87^ who note that semilocal DFT nearly always overestimates the high-frequency dielectric response, while hybrid functionals nearly always underestimate the high-frequency dielectric response, with PBE0 performing worse than HSE06. Considering the tetrahedral zincblende structure of CuI, it is reasonable to assume that similar trends are followed here. Due to the fact that the dielectric constant is quite small for CuI, these quite large percentage errors in ϵ_0_ have significant effects on the POP and IMP scattering rates, as shown in Figure S4 in the Supporting Information. To this end, the dielectric constant used in the AMSET calculations in Figures 4 and 5 is that determined from experiment (6.5, where ϵ_ionic_ is set as the calculated value of 1.1 from DFPT, and the remainder is assigned to ϵ_∞_), with lower and upper limits (5.27 and 8.85) shown for context in the Supporting Information. More sophisticated methods for calculating the dielectric response that can accurately model excited state features, such as *GW* (Green’s function *G* with screened Coulomb interaction *W*) and BSE (Bethe–Salpeter equation) implementations, would provide a clearer picture,^88^ but exceed the scope of the current work.

**Table 2:** Dielectric Constants Calculated Using a Variety of Methods^a^

details	ϵ_ionic_	ϵ_∞_	ϵ_0_
PBEsol DFPT; PBEsol IP-RPA*	1.10	7.75	8.85
PBEsol DFPT; PBE0 IP-RPA*	1.10	4.17	5.27
PBE0 FD; PBE0 IP-RPA*	1.65	4.17	5.82
materials project: PBE	0.87	6.82	7.69
Li et al.^89^ PBE	1.53	4.77	6.30
Hanson et al. (exp.)^86^			6.5

a * denotes calculations from this work; DFPT (density functional perturbation theory) or FD (finite differences method) calculations were used to determine the low-frequency, ϵ_ionic_, response, while IP-RPA (independent particle random phase approximation) optical calculations were used to determine the high-frequency, ϵ_∞_, response. Further details can be found in the methodology section.

#### Combined Approach

While AMSET correctly predicts the relative importance of the different scattering mechanisms, the low concentration regime phonon-limited mobility predicted by AMSET and the IBTE method varies by a factor of 4. As POP scattering is the primary mobility-limiting mechanism in this material at low carrier concentrations, this overestimation of the scattering rate results in mobility values that are lower than those reported in the experimental literature (notably the single crystal result from Chen et al.).^41^ One way to explain this is the inelastic treatment of POP in AMSET. Indeed, AMSET approximates POP scattering using the self-energy relaxation time approximation (SERTA), which as part of its formalism considers scattering only in the forward direction relative to charge carrier motion. The omission of backward scattering can lead to an underestimation of phonon-limited mobility, the extent of which varies between materials depending on the complexity of the band edge (single band or multidegenerate), the effective mass, and spin–orbit effects.^65,90^

The comparison between phonon-limited mobility and experimental results can be challenging since most calculations do not consider impurities or defects. While SERTA serves as an approximation of IBTE, it is advisible to prioritize the latter due to its comprehensive nature, even if certain SERTA results appear closer to experimental data. For instance in Si, the computed electron mobility with the two methods fall within the range of experimentally reported mobilities,^64^ although the hole mobility is overestimated by both approaches—only when performing simulations using the experimental lattice parameter and fitting the band structure to the experimental hole effective mass is agreement with experimental mobility recovered using the IBTE approach;^90^ for SiC the IBTE performs better than the SERTA for electron mobility (SERTA underestimates by around 30%), while they straddle the upper and lower side of experimental results for the hole mobility, respectively;^91^ and for GaAs both the implementations provide rather unreliable results, not helped by the large range of experimental mobilities reported.^92^ Surveying over 50 materials, Claes et al. show that differences in computed mobility using IBTE and SERTA can reach up to 60%,^65^ and that the differences are often particularly large for binary halides with low ω_POP_ such as NaI, CsI, and TlBr. The general trend is that SERTA will, if at all, underestimate phonon-limited mobilities compared with the exact IBTE results. Crucially, it is difficult to predict a priori the severity of the underestimation.

To this end, mobility values obtained using SERTA under ABINIT are compared with those acquired using AMSET, as illustrated in Figure S3. It is found that the agreement between the two SERTA results is close, and this indicates that POP scattering is potentially overestimated in AMSET for CuI due to the SERTA. Therefore, we propose that the mobility limit in CuI is determined in the low concentration regime by IBTE e–ph scattering and in the high concentration regime by ionized impurity scattering.

Figure 5 shows the hole mobility determined by summing the e–ph contribution from IBTE and the IMP contribution from AMSET assuming the validity of Matthiessen’s rule on mobility . While this does not represent an exact solution to the BTE, it provides a reasonable upper limit to the mobility that is achievable in CuI. The e–ph IBTE and total AMSET drift mobilities (computed using the experimentally determined dielectric constant of 6.5) are also shown, as are experimental Hall mobility values from the literature. As the Hall factor (*r*_H_) is close to 1 for CuI and CuBr,^93,94^ it is reasonable to compare the simulated drift mobility with experimental Hall mobility (*r*_H_μ ∼ μ_H_).

The combined mobility from IBTE and IMP acts as an upper limit to hole mobility in CuI, within which the presently available data from experiment falls. First, single crystal data are considered (orange stars in Figure 5), which should represent the highest achievable experimental mobility and truest comparison to simulation, as only the intrinsic, material-dependent scattering processes should be present. It is well-known however that single crystal size, cleanliness, and surface defects can all impact mobility measurements. Record single crystal mobility is reported by Chen et al.,^41^ achieving a value of 43.9 cm^2^ V^–1^ s^–1^ at a carrier concentration of 4.3 × 10^16^ cm^–3^ in a sample of dimensions 15 × 10 × 1 mm. This is in the electron–phonon scattering limit and suggests significant scope for improvement in mobility at low carrier concentrations. Other single crystals have been synthesized by Lv et al.,^42^ obtaining a sample of similar size and carrier concentration but with a reduced mobility of 12.8 cm^2^ V^–1^ s^–1^, and Matsuzaki et al.,^97^ achieving mobility up to 29 cm^2^ V^–1^ s^–1^ at low concentrations (2.0 × 10^14^ cm^–3^) and 19 cm^2^ V^–1^ s^–1^ at carrier concentrations approaching the degenerate conductivity limit. By considering the POP scattering rate from AMSET as the dominant low concentration scattering mechanism, Chen’s measurement exceeds the predicted mobility. It is reasonable to assume that this single crystal measurement is within the “low” carrier concentration regime, as a rough calculation of the Mott criterion using the parabolic (transport) band edge effective masses and the experimental dielectric constant indicates a carrier density of 8.5 × 10^17^ cm^–3^ (1.65 × 10^18^ cm^–3^). This failure of the SERTA to describe the low carrier density experimental data points was the first indication that a more sophisticated treatment of the e–ph scattering would be required for CuI.

Turning now to thin films, the record mobility measurement is more difficult to identify. Several papers report promising mobilities, even surpassing the 43.9 cm V^–2^ s^–1^ from Chen et al.: 35 cm^2^ V^–1^ s^–1^ at 8.5 × 10^18^ cm^–3^ via liquid iodization of metallic Cu from Wang et al.;^99^ 35 to 50 cm^2^ V^–1^ s^–1^ at concentrations in the region of 1 × 10^18^ cm^–3^ via iodization of metallic Cu on crystalline Si substrates from Madkhali et al.;^100^ and 110 cm^2^ V^–1^ s^–1^ at 1.1 × 10^18^ cm^–3^ via MBE on crystalline Si from Ahn et al.^101^ However, these measurements are from extremely thin films that have XRD peaks that can be attributed to the Cu and c-Si substrates, which themselves are extremely good charge carriers, making it difficult to decouple the transport properties of the substrate from those of the film. These results are therefore omitted from Figure 5. Storm et al. report consistently high thin film mobility via pulsed laser deposition (PLD, purple triangles in Figure 5) over a wide range of carrier concentrations,^38,40^ which comprises the most reliable thin film data available in the literature. At the higher end of the carrier concentrations reported, films deposited from sputtering and liquid iodization appear to approach the mobility limit arising from ionized impurity scattering, peaking around 10 cm^2^ V^–1^ s^–1^. Across the entire carrier concentration range, the experimental results fall within the IBTE + IMP limit (and critically fall outside the SERTA + IMP, i.e., AMSET, limit), justifying our approach.

The final and perhaps most crucial point to consider is the electronic performance achievable via scalable synthesis methods. While PLD, MBE and sputtering offer reasonably high mobilities across the carrier concentration range, they are not commercially viable deposition techniques. Instead, inkjet printing, spin coating, and, potentially, iodination reactions are likely to be the most industrially relevant. The hole mobilities reported from these methods seldom exceed 10 cm^2^ V^–1^ s^–1^ regardless of carrier concentration, owing to the lower crystallinity of the films and subsequent grain boundary and interface scattering. Such CuI films are roughly on par with SnO mobility,^15^ but are beaten by Ba_2_BiTaO_6_ (30 cm^2^ V^–1^ s^–1^)^19,20^ and the layered oxychalcogenide (Cu_2_S_2_)(Sr_3_Sc_2_O_5_) (150 cm^2^ V^–1^ s^–1^),^102^ and are still up to 2 orders of magnitude lower than the degenerately doped n-type transparent conductors (In_2_O_3_ 130 cm^2^ V^–1^ s^–1^, Ga_2_O_3_ 75 cm^2^ V^–1^ s^–1^, SnO_2_ 130 cm^2^ V^–1^ s^–1^, BaSnO_3_ 320 cm^2^ V^–1^ s^–1^, and ZnSb_2_O_6_ 49 cm^2^ V^–1^ s^–1^).^103−107^ Despite this, CuI has been successfully used to make thin film transistors via solution-based and inkjet deposition with competitive switching ratios and current densities,^37,43,108^ and amorphous CuI thin-film transistors (TFTs) have been reported to outperform polycrystalline devices.^109^ Such applications often require a carrier density lower than 10^20^ cm^–3^, where the mobility of CuI can be significantly improved. The prediction of such a large scope for improvement in CuI mobility indicates that other scattering processes may also be in play, such as surface and grain boundary scattering, which could be mitigated as deposition and device engineering process become more sophisticated. These results indicate that CuI will retain its position as the front-runner in the race for a marketable p-type TCM, and provide useful insights for quality control in CuI.

### Defect Chemistry

**Figure 6:**
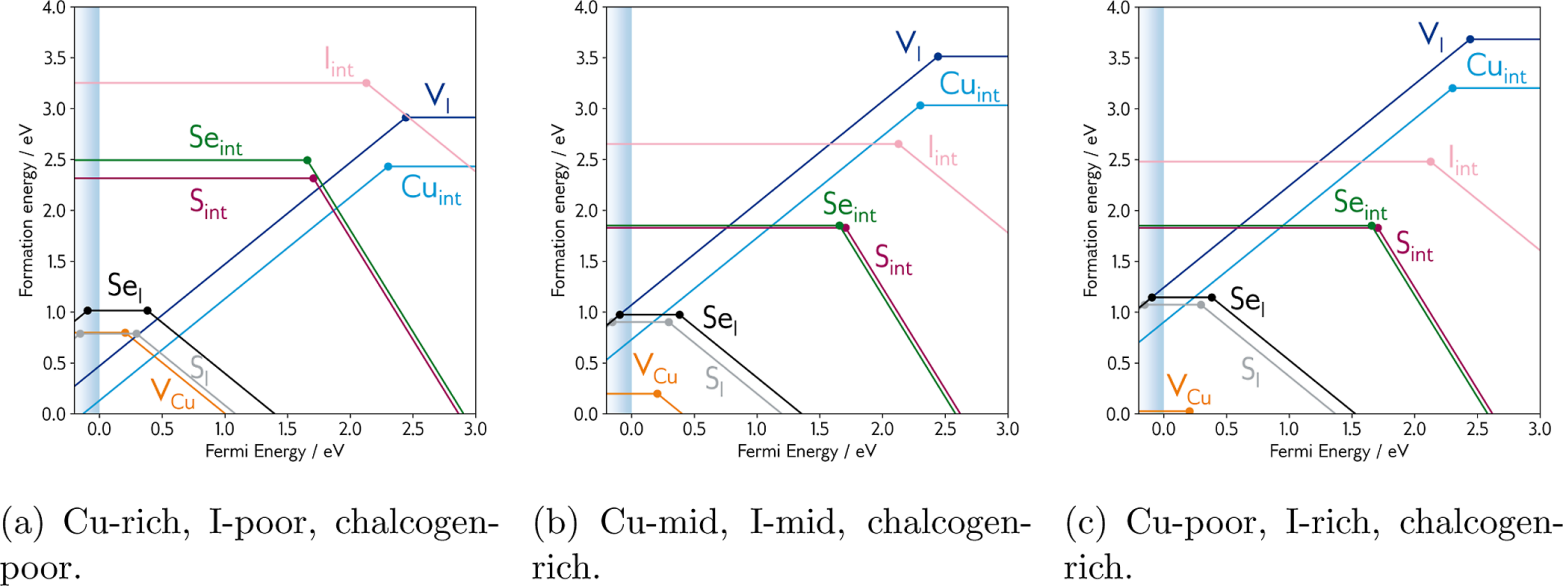
Transition level diagrams for CuI under three sets of chemical potentials. Fermi energy (eV) on the *x*-axis, formation energy (eV) on *y*-axis. The valence band maximum (VBM) is denoted by the shaded blue region. The gradient of each line represents the charge state, and filled circles denote transition levels where two charge states are in thermodynamic equilibrium. (a) Cu-rich, I-poor, chalcogen-poor. (b) Cu-mid, I-mid, chalcogen-rich. (c) Cu-poor, I-rich, chalcogen-rich.

Suitable point defect chemistry is crucial to the operation of transparent conducting materials. For a p-type system, it is necessary to have low formation energy acceptor defects at Fermi levels close to, or ideally within, the VBM. As the separation between the defect state and band edge decreases, more holes can be generated at the VBM through thermal excitation of an electron to the defect (with energy ∼ *k*_B_*T*), increasing conductivity. It is also desirable that such p-type defects generate delocalized charge density, enabling a high charge carrier mobility with no energetic barrier—the formation of localized charge, or hole polarons, necessitates an activation energy *E*_A_ (with an Arrhenius-like temperature dependency) for charge transport, which significantly reduces optoelectronic performance (consider the polaron-limited mobility in CuAlO_2_ of 3 cm^2^ V^–1^ s^–1^).^7,110^ To this end, both the intrinsic defect chemistry of CuI and the effects of chalcogen doping (S and Se) were studied using hybrid functionals. Transition level diagrams under a variety of Cu–I chemical potentials (Cu-rich, midpoint, and Cu-poor) are shown in Figure 6, while tabulated formation energies of defects and competing phases, along with extended methodology, can be found in the Supporting Information.

#### Intrinsic Defects

The copper vacancy is the predominant defect in CuI, with a formation energy below 1 eV regardless of the growth conditions. It has a transition level from the 0/1—charge state approximately at 0.24 eV from the band edge, indicative of a deep defect. A significant disruption to the local crystal structure upon vacancy formation supports this behavior, with one nearby Cu atom relaxing toward the vacancy by 0.09 Å. The hole generated at the VBM by the formation of a copper vacancy is found to localize on this displaced atom, shown in Figure S6. However, analysis of the Fröhlich polaron constant for CuI (α = 0.44 for PBEsol and 1.55 for PBE0) indicates that such a polaron is likely to be spread over several unit cells (supported by observation of several metastable defect geometries within 1 meV of the ground state with less localized hole density, see Figures S5 and S6) so should still result in reasonable charge carrier mobility, and crucially indicates that the band carrier model assumed for the charge transport calculations is still valid. Under Cu-poor conditions, charge carrier concentrations of up to 2 × 10^19^ cm^–3^ are predicted solely due to the increased concentration of copper vacancies, indicating that this species is the key factor in controlling p-type conductivity in CuI.

The remaining intrinsic defect species are the p-type iodine interstitial (I_int_), the n-type iodine vacancy (V_I_), and the n-type copper interstitial (Cu_int_). The iodine interstitial is an ultradeep acceptor with prohibitively high formation energies under all growth conditions (2.5 eV in the best case), and is unlikely to play a major role in boosting p-type conductivity. The n-type defects are both deep donors and under Cu-rich conditions are predicted to charge compensate the holes generated by the copper vacancy (where the blue and orange lines intersect in Figure 6a). However, as growth conditions are modulated toward a Cu-poor environment, the formation energies of both the iodine vacancy and copper interstitial are sufficiently raised such that they can no longer charge compensate.

#### Extrinsic Defects—Post Hoc Ergo Propter Hoc?

Chalcogen (S, Se) doping has been explored as a route to further enhance hole conductivity both computationally and experimentally.^30,38,40,68^ The formation energies of Cu_*x*_S_*y*_ and Cu_*x*_Se_*y*_ competing phases within the chemical potential limits of CuI control the chemical potentials of the dopant atoms, but it is observed that synthesis conditions have little effect on the formation energies of the substitutional species S_I_ and Se_I_ due to the concomitant changing chemical potential of iodine. Much larger changes in formation energy are observed for the interstitial defects S_int_ and Se_int_, where the changing chemical potential of iodine has no impact on the formation energy of the chalcogen defect.

**Figure 7:**
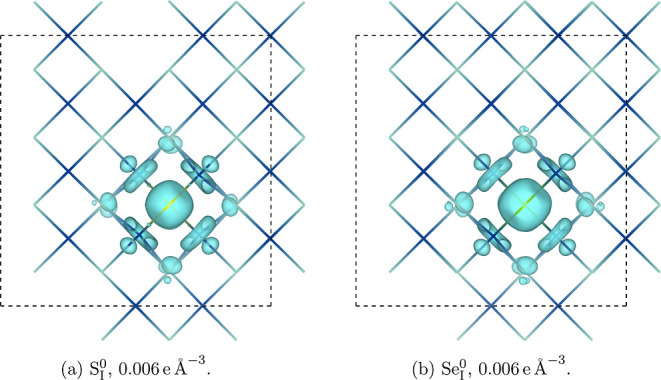
Partial hole density generated by various defects in CuI. Defect supercell is denoted by a dotted black line; blue regions are Cu, green regions are I, and lime green regions are dopants. (a) S_I_^0^, 0.006 e Å^–3^. (b) Se_I_^0^, 0.006 e Å^–3^.

The substitutional defects themselves are higher in energy than the copper vacancy under p-type growth conditions (Figure 6b,c), at around 0.9 eV for S_I_ and 1.0 eV for Se_I_, and have deeper transition levels. This suggests a greater degree of charge localization than for the copper vacancy, which is shown in Figure 7. The hole generated by the formation of both defects is strongly bound to the site of the substitution and causes a significant inward relaxation of the four surrounding copper atoms. This deep transition level is supported by the experimental work of Storm et al., who report a binding energy for the Se substitution of around 300 meV.^40^ Graužinytė et al. predict shallower transition levels for both S_I_ and Se_I_, but these are from defect relaxations performed with a semilocal exchange–correlation functional without SOC, leading to an overdelocalization of charge and an incorrect defect geometry.^68^

Finally, the interstitial chalcogen species are much higher in formation energy and are predicted to act as ultradeep acceptors, with negative-*U* 0/2—transitions occurring at Fermi levels close to 2.0 eV. These characteristics preclude them from having any significant effect on the electronic properties of CuI.

Overall, the defect chemistry of CuI is rather straightforward. Native p-type conductivity is enabled by the formation of copper vacancies, the concentration of which can be fine-tuned by strict control of the copper chemical potential during synthesis, while the remaining intrinsic species have little to no effect on the defect landscape. Despite the small improvements in electronic properties after doping with S and Se reported in the literature, these dopants are predicted to be electronically inactive with deep transition levels that result in significant charge trapping. This is an example of the logical fallacy post hoc, ergo propter hoc, or “after this, therefore because of this”. A number of factors could contribute to the prediction of deeper transition levels in the current work compared to those from Graužinytė et al.: a more thorough exploration of the potential energy surface for each defect state via use of the ShakeNBreak method; full geometric optimization with hybrid functionals, rather than relaxation with semi-local functionals plus a hybrid functional single-shot on a volume-scaled structure; and the explicit inclusion of SOC during defect calculations to accurately model the position of the band edges. With these new insights on the electronic role of chalcogen dopants, it is instead proposed that indirect changes in host chemical potentials as a result of chalcogen doping have a greater impact on overall hole concentration. Furthermore, effects on crystallinity and morphology should be considered during experimental investigation of chalcogen doping, as this can be a further source of indirect improvements to optoelectronic performance.

## Conclusions

This article examined the suitability of CuI as a high performance p-type transparent conductor. Using sophisticated charge transport modeling, an upper limit to hole mobility as a function of carrier concentration was predicted, ranging from 162 cm^2^ V^–1^ s^–1^ in the phonon-limited range to 32.6 cm^2^ V^–1^ s^–1^ in the degenerately doped, ionized impurity-limited range. These results suggest a significant scope for improvement in experimental mobility, particularly in the low to mid concentration range, which could be achieved by mitigating surface and grain boundary scattering processes and by further optimization of synthesis conditions. The prospect of achieving samples with mobilities that closely align with our theoretical projections holds significant promise. Such progress has the capacity to yield a noteworthy 2- to 3-fold enhancement in the existing experimental FoM, Φ, for CuI, thereby propelling it to a competitive stance alongside the finest n-type materials currently prevalent on the market. An examination of the defect chemistry revealed that the copper vacancy is the predominant source of charge carriers in CuI and that modulation of the copper chemical potential during synthesis is key to controlling the hole concentration. The chalcogens S and Se are predicted to be electronically inactive dopants but can result in increased conductivity and hole concentration via indirect effects such as improved sample crystallinity. Revisiting nondoped CuI and exercising greater control over the copper vacancy could lead to enhanced mobility at lower carrier concentrations, which would improve the performance of CuI in thin film electronics. This work will act as a useful benchmark to experimental studies on CuI and related p-type transparent conductors and indicates that there is still scope for enhanced optoelectronic performance.

## Supporting Information Available

Phonon dispersion, charge transport constants, further AMSET plots, and convergence testing (PDF)

## Notes

Online repository which can be found at DOI: https://doi.org/10.5281/zenodo.8318329 contains charge transport and defect formation energy data.

## Footnotes

a Generally, the figure of merit is calculated as the ratio between electrical conductivity σ and the visible absorption coefficient α, where *T*_vis_ is transmission and *R*_s_ is the sheet resistance, related to conductivity and thickness *d*.
