# Supplementary material for: Limits to Hole Mobility and Doping in Copper Iodide
Source: Chem Mater. 2023 Oct 25;35(21):8995–9006. doi: 10.1021/acs.chemmater.3c01628 (PMC10653089; doi:10.1021/acs.chemmater.3c01628)
Supplement: Supplementary file 1 — cm3c01628_si_001.pdf [file cm3c01628_si_001.pdf]

Limits to Hole Mobility and Doping in Copper Iodide

Joe Willis,[†,‡,⊥] Romain Claes,[¶,⊥] Qi Zhou,[†,‡] Matteo Giantomassi,[¶] Gian-Marco Rignanese,[¶] Geoffroy Hautier,[*,¶,§] and David O. Scanlon[*,†,‡,∥]

†*Department of Chemistry, University College London, 20 Gordon Street, London, WC1H 0AJ, UK.*

‡*Thomas Young Centre, University College London, Gower Street, London, WC1E 6BT, UK.*

¶*UCLouvain, Institute of Condensed Matter and Nanosciences (IMCN), Chemin des Étoiles 8, B-1348 Louvain-la-Neuve, Belgium.*

§*Thayer School of Engineering, Dartmouth College, Hanover, New Hampshire 03755, USA.*

∥*School of Chemistry, University of Birmingham, Edgbaston, Birmingham B15 2TT, United Kingdom*

⊥*These authors contributed equally to this work.*

E-mail: geoffroy.hautier@dartmouth.edu; d.o.scanlon@bham.ac.uk

Supplementary Information contains phonon dispersion, charge transport constants, further AMSET plots and some convergence testing. Online repository, which can be found at DOI: `https://doi.org/10.5281/zenodo.8318329` contains charge transport and defect formation energy data.

Supplementary Information

Phonon dispersion curves

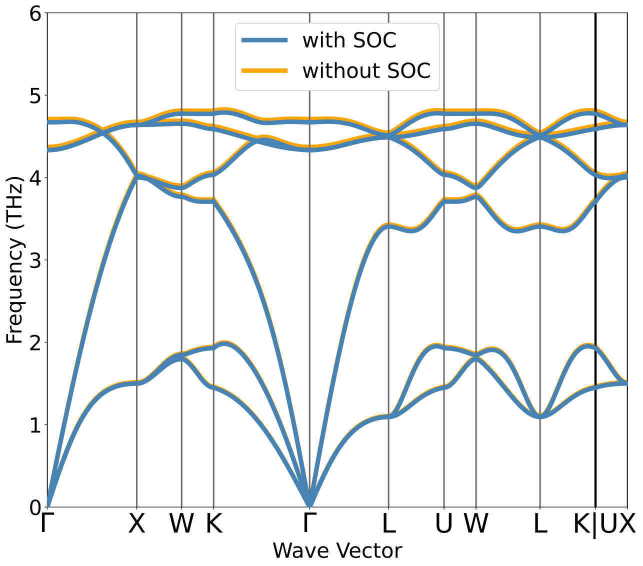

Figure S1: Phonon dispersion curves for CuI with and without SOC.

Charge Transport

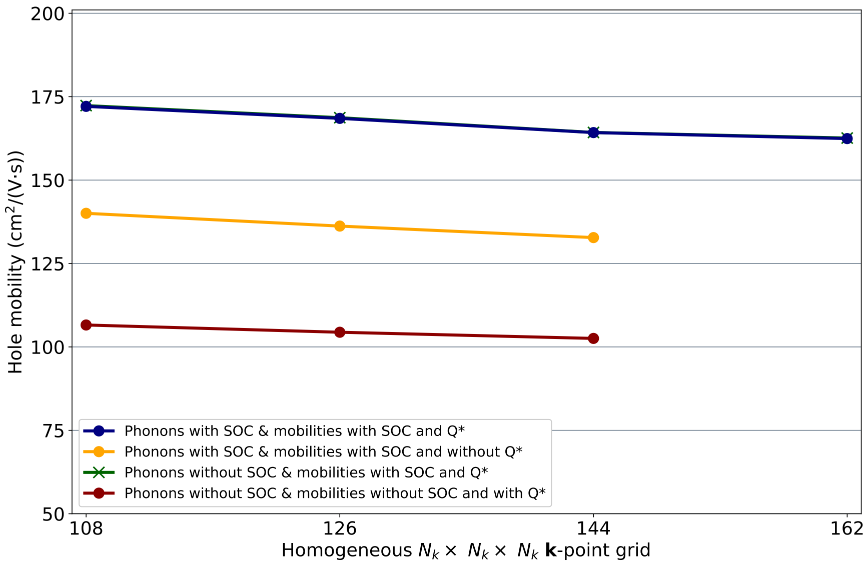

Figure S2: IBTE hole mobility in CuI as a function of the **k**- and **q**-point grids at 300K using different treatments (the density of **q**-points is the same as the density of **k**-points). Due to their long-range nature, quadrupolar interactions must be taken into account for an accurate description of the scattering potential and mobilities.[1,2] SOC also plays an important role in the transport properties of CuI and must also be taken in account. In this plot, SOC+Q* mobilities obtained with SOC phonons, SOC+noQ* mobilities with SOC phonons, SOC+Q* mobilities obtained with noSOC phonons and noSOC+Q* mobilities obtained with noSOC phonons are represented in blue, orange, green and red, respectively. The absence of SOC in the phonon calculations does not affect the mobility results, as the blue and green curves overlap.

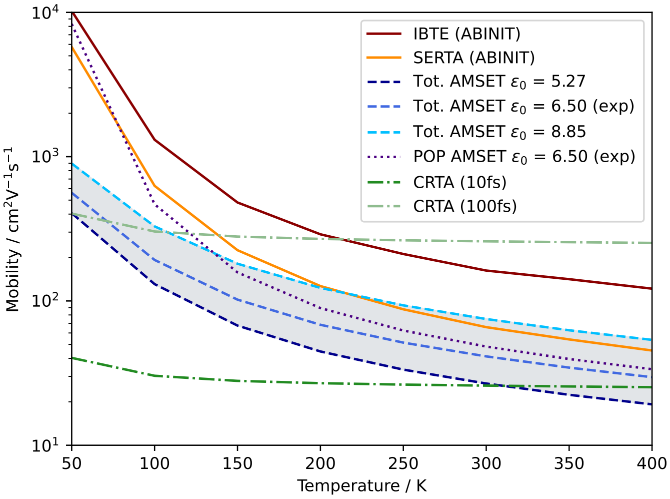

Figure S3: CuI hole mobility against T computed with different methods. This graph shows the influence of dielectric constants on the results obtained with AMSET but also demonstrates the effect of the use of SERTA in the calculation of POP with AMSET whose results are close to the results obtained with this same approximation in ABINIT.

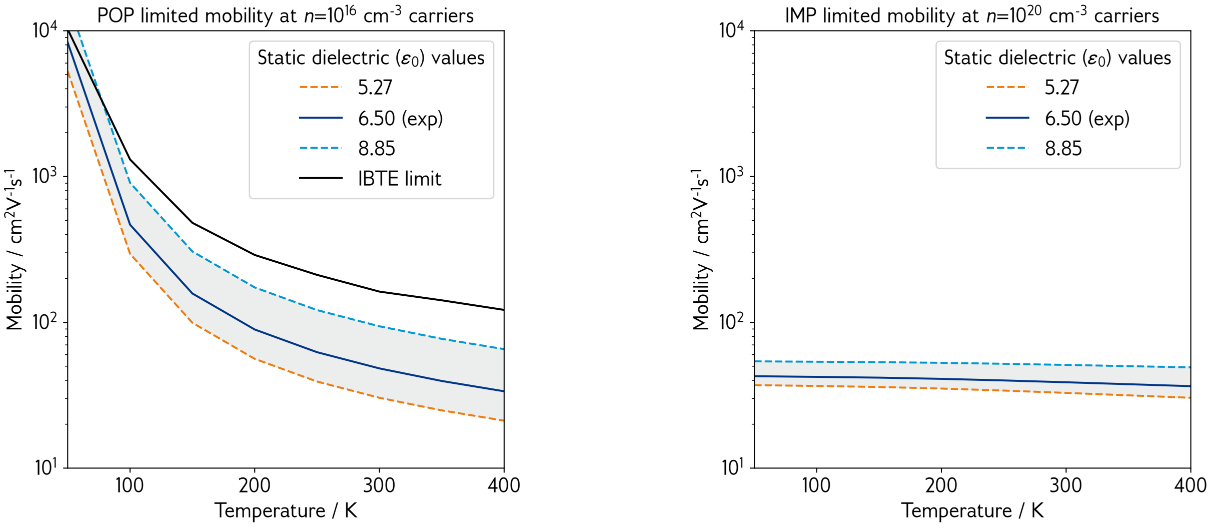

(a) Low carrier concentration, $1\times10^{16}\,\mathrm{cm}^{-3}$.

(b) High carrier concentration, $1\times10^{20}\,\mathrm{cm}^{-3}$.

Figure S4: CuI hole mobility as a function of temperature at two carrier concentrations showing the dominant scattering mechanism and the dependence on the value of ϵ_0.

Defects

The ShakeNBreak method was used to identify the lowest energy structures for point defects in CuI.[3,4] Upon generation of a defect, a series of targeted bond distortions (the quantity and direction of which are determined using simple valence chemistry rules) is applied, followed by a small, random "rattling" of the structure, in order to break the local symmetry. These distorted structures, along with the "unperturbed" starting structure, are then relaxed using coarse calculation parameters to get a relatively cheap sampling of the complex defect potential energy surface. The lowest energy structure identified from this initial search is then relaxed using full accuracy parameters. This method allows the search for a defect ground state to begin from several, distinct points on the potential energy surface, decreasing the probability of getting stuck in a high energy local minimum.

Copper vacancy

Several metastable structures with energy less than 1 meV difference from the structure used to plot the transition level diagrams were identified using the ShakeNBreak method. Their relative energies are shown in Figure S5. The charge localisation is different between the structures, with Figure S6 showing the difference between the 'unperturbed" (no distortions) and lowest energy (−50% bond distortion) structures. The energy difference is so small that at finite temperature, it is likely that the real charge density is some combination of the two, indicating a reasonably delocalised charge density (as stated in the main text).

Iodine vacancy

The iodine vacancy was investigated in the charge states 1−, 0 and 1+, following reports from previous computational studies of a $1+/1-$ transition level.[5] The ground state of the neutral vacancy is found by a 20% bond distortion, shown in Figure S7a, and sees an opposite pair of Cu atoms relax into the vacancy, while another pair relax away from it. The structure obtained from the "unperturbed" relaxation is significantly different, shown

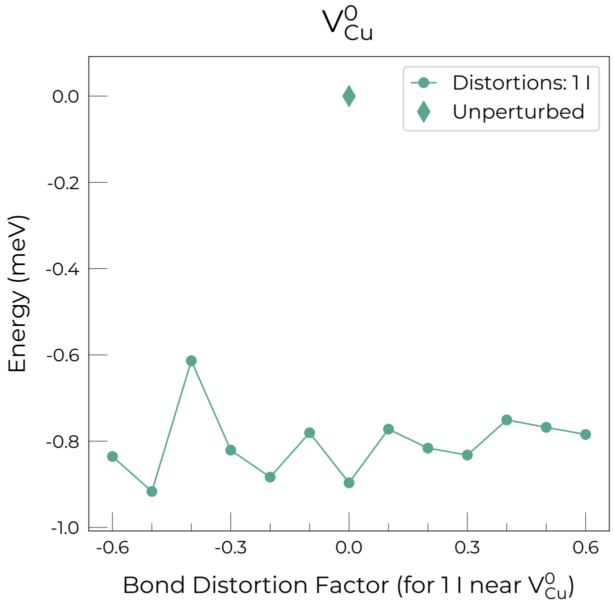

Figure S5: Energy profile of relaxed copper vacancies with various bond distortions.

in Figure S7b, consisting of an open cage around the vacancy, and then migration of the remaining electron to an adjacent I atom, subsequently causing an inward relaxation of four Cu atoms, and is 100 meV higher in energy. It is possible that this higher energy structure is the one identified by previous studies, pushing the neutral defect higher in energy on the transition level diagram and facilitating the negative-U behaviour. The fully ionised ground state iodine vacancy is shown in Figure S7c, and is a simple outward relaxation of the Cu atoms around the vacancy, caused by the removal of a centre of negative charge.

This example demonstrates the importance of sampling the potential energy surface thoroughly, although admittedly for the iodine vacancy the formation energies are so high that these differences in defect geometry play little to no rôle in the overall defect chemistry of CuI.

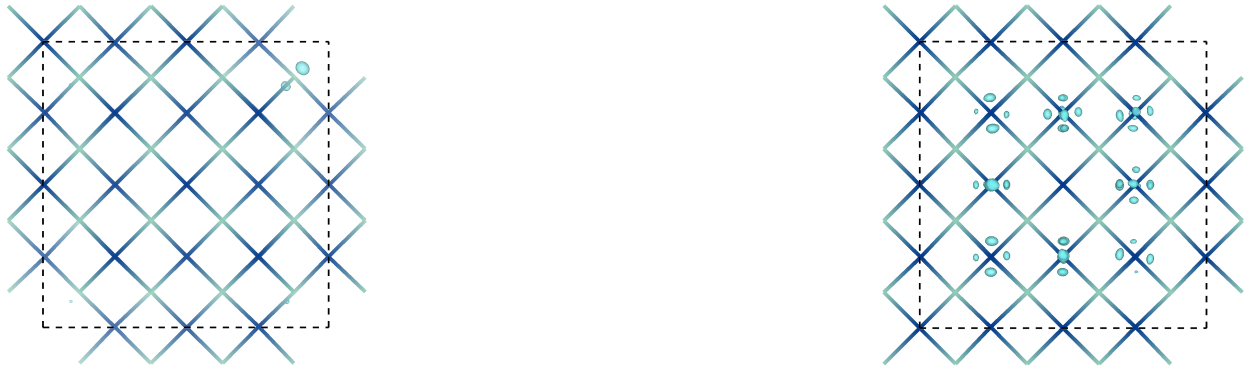

(a) Neutral ground state copper vacancy (-50% BDM).

(b) Neutral meta-stable copper vacancy ("unperturbed").

Figure S6: Geometries and partial charge density of the copper vacancy.

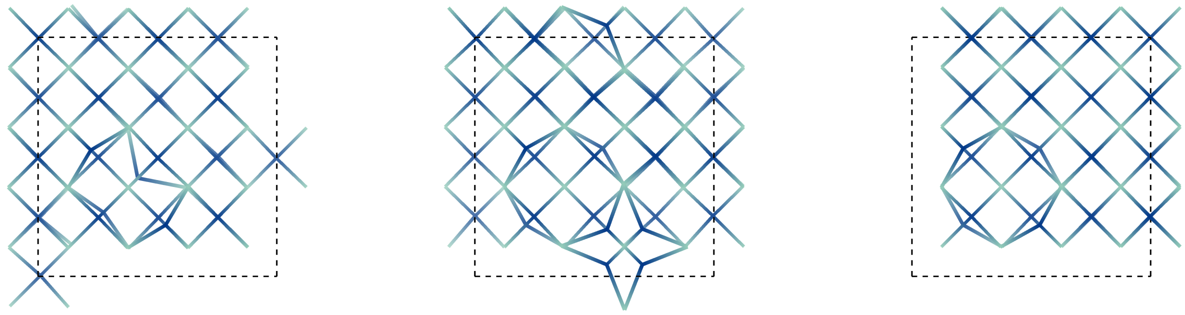

(a) Neutral ground state iodine vacancy (20% BDM).

(b) Neutral meta-stable iodine vacancy.

(c) Fully ionised ground state iodine vacancy.

Figure S7: Geometries of the iodine vacancy.

CuI Materials Properties

This section contains the calculated values of each of the properties used for AMSET calculations, as determined using the PBEsol and PBE0 functionals.

Polar optical phonon frequency PBEsol: 4.43 THz

Polar optical phonon frequency PBE0: 3.87 THz

Elastic constant PBEsol DFPT:

$$\begin{bmatrix} 63.0 & 43.3 & 43.3 & 0 & 0 & 0 \\ 43.3 & 63.0 & 43.3 & 0 & 0 & 0 \\ 43.3 & 43.3 & 63.0 & 0 & 0 & 0 \\ 0 & 0 & 0 & 23.8 & 0 & 0 \\ 0 & 0 & 0 & 0 & 23.8 & 0 \\ 0 & 0 & 0 & 0 & 0 & 23.8 \end{bmatrix}$$

Piezoelectric constant PBEsol DFPT:

$$\begin{bmatrix} 0 & 0 & 0 & 0.10 & 0 & 0 \\ 0 & 0 & 0 & 0 & 0.10 & 0 \\ 0 & 0 & 0 & 0 & 0 & 0.10 \end{bmatrix}$$

High-frequency dielectric constant PBE0 optics:

$$\begin{bmatrix} 4.17 & 0 & 0 \\ 0 & 4.17 & 0 \\ 0 & 0 & 4.17 \end{bmatrix}$$

Low-frequency dielectric constant PBEsol DFPT:

$$\begin{bmatrix} 1.10 & 0 & 0 \\ 0 & 1.10 & 0 \\ 0 & 0 & 1.10 \end{bmatrix}$$

Low-frequency dielectric constant PBE0 FD:

$$\begin{bmatrix} 1.65 & 0 & 0 \\ 0 & 1.65 & 0 \\ 0 & 0 & 1.65 \end{bmatrix}$$

Total (static) dielectric constant PBEsol DFPT & PBE0 optics:

$$\begin{bmatrix} 5.27 & 0 & 0 \\ 0 & 5.27 & 0 \\ 0 & 0 & 5.27 \end{bmatrix}$$

Total (static) dielectric constant PBE0 FD & PBE0 optics:

$$\begin{bmatrix} 5.82 & 0 & 0 \\ 0 & 5.82 & 0 \\ 0 & 0 & 5.82 \end{bmatrix}$$

Convergence

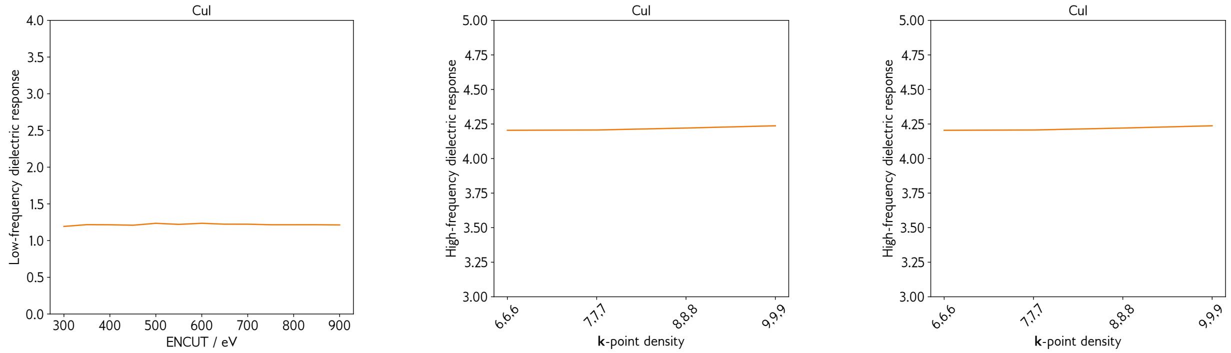

(a) DFPT plane-wave cut-off.

(b) DFPT **k**-points.

(c) Optics **k**-points.

Figure S8: Convergence of the low-frequency dielectric response against plane-wave energy cut-off for the copper halides.

References

(1) Brunin, G.; Miranda, H. P. C.; Giantomassi, M.; Royo, M.; Stengel, M.; Verstraete, M. J.; Gonze, X.; Rignanese, G.-M.; Hautier, G. Electron-phonon beyond Fröhlich: dynamical quadrupoles in polar and covalent solids. *Phys. Rev. Lett.* **2020**, *125*, 136601.

(2) Brunin, G.; Miranda, H. P. C.; Giantomassi, M.; Royo, M.; Stengel, M.; Verstraete, M. J.; Gonze, X.; Rignanese, G.-M.; Hautier, G. Phonon-limited electron mobility in Si, GaAs, and GaP with exact treatment of dynamical quadrupoles. *Phys. Rev. B* **2020**, *102*, 094308.

(3) Mosquera-Lois, I.; Kavanagh, S. R.; Walsh, A.; Scanlon, D. O. Identifying the ground state structures of point defects in solids. *npj Computational Materials* **2023**, *9*, 25.

(4) Mosquera-Lois, I.; Kavanagh, S. R.; Walsh, A.; Scanlon, D. O. ShakeNBreak: Navigating the defect configurational landscape. *Journal of Open Source Software* **2022**, *7*, 4817.

(5) Matsuzaki, K.; Tsunoda, N.; Kumagai, Y.; Tang, Y.; Nomura, K.; Oba, F.; Hosono, H. Hole-Doping to a Cu(I)-Based Semiconductor with an Isovalent Cation: Utilizing a

Complex Defect as a Shallow Acceptor. *Journal of the American Chemical Society* **2022**, *144*, 16572–16578.
